# Nurses' Perceptions on How Recovery-Oriented Mental Health Care Can Be Developed and Implemented

**DOI:** 10.1155/2023/4504420

**Published:** 2023-08-23

**Authors:** Kebope Mongie Kealeboga, Mofatiki Eva Manyedi, Salaminah Moloko-Phiri

**Affiliations:** ^1^University of Botswana, Faculty of Health Sciences, School of Nursing Science, Gaborone, Botswana; ^2^North-West University Faculty of Health Sciences, School of Nursing Science, Mafikeng, South Africa

## Abstract

**Aim:**

This study explored how nurses working in inpatient mental health units perceived the development and implementation of a recovery-oriented mental healthcare programme (ROMHCP).

**Background:**

The recovery-oriented mental healthcare approach (ROMHCA) in mental health is regarded as the future of mental health services and has been implemented in different countries worldwide. However, regarding developing and implementing the recovery approach, Africa appears to have been left behind by the rest of the continents.

**Design:**

The study used a qualitative approach to describe how a recovery-oriented mental healthcare approach could be developed.

**Methods:**

Thirty nurses who worked in Botswana's four inpatient mental health facilities consented and voluntarily participated in the study. Data were collected from February to mid-March 2022 through online focus group discussions and analysed using thematic analysis. The COREQ checklist was used to report the findings.

**Results:**

Two main themes emerged as follows: (i) developing and implementing a recovery-oriented mental healthcare programme is possible and (ii) certain elements are required to develop and implement ROMHCP.

**Conclusion:**

The participants believed that people diagnosed with mental illness could recover from the illness and suggested how it could be achieved. They also contended that the programme's success would lie mainly with multisectoral support from policymakers, facilities, hospital personnel, patients, and the community. *Clinical Relevance*. ROMHCP has the potential to benefit people with mental illness in the country. In addition, it would allow nurses to improve their knowledge and skills in managing mental illnesses. *Patient or Public Contribution*. The patients and the general public did not contribute to the study's concept, design, and outcomes. However, the nurses working in mental health facilities volunteered to participate in the study.

## 1. Introduction

Mental health services, notably the community based, from certain Western high-income countries have embraced ROMHCA as a new dimension for mental health departments when treating people diagnosed with mental illness [1] online [2, 3]. The recovery vision entails rethinking and reforming the current mental healthcare practice, whose approach is predominantly biomedical, to a person-centred approach in providing care to people with mental illness. The biomedical approach in mental health positions the cause of mental illness to brain disorders, thus relating the cause of mental illness to biological abnormalities in an individual, and therefore, one will need pharmacological interventions [4]. Doing so has led to shifting to ROMHCA. ROMHCA recognises the individuality of a person diagnosed with a mental illness. It is entrenched in the principles of self-determination, empowerment, citizenship, love, connectedness, respect, and the installation of hope for the future of people with mental illness [5, 6]. In this model, the patient drives their recovery process.

There is no common understanding of what mental health constitutes. Anthony [7] gave the most popular and widely accepted definition. He explained recovery as living a gratifying life notwithstanding being decapacitated by the symptoms of mental illness. Ørjasæter and Almvik [8] also described recovery as a multifaceted process through which individuals with mental illness live satisfying and fulfilling lives like any other citizen. This definition validates the personal nature that people attribute to recovery. The literature has revealed that the person centeredness of recovery alone highlights the need for individualised care plans for people with mental illness [9, 10].

Intervention programmes have been developed to facilitate ROMHCA. For instance, in the United States of America, Copeland [11] developed a Wellness Recovery Action Plan (WRAP) programme to teach healthcare workers and people with mental illness how to live with and manage mental illness. WRAP is a framework where healthcare workers use to learn more about mental illness and how to manage it from a recovery-oriented approach, and it has been proven to improve the general well-being of people with mental illness [12]. In England, Bird and Slade [13] developed a REFOCUS intervention to improve the working relationships between the staff and patients. REFOCUS is an intervention focusing on activities promoting recovery and recovery-working practices and relationships among staff for them to connect better with patients [14]. In addition, it coaches staff to understand mental health users' values and treatment preferences to support them in a better recovery process [14].

Flaherty-Jones et al. [15] developed a Steps to Recovery (STR) programme in Australia to improve mental illness by providing information, including instilling hope for the future by providing people with mental illness skills on managing mental illnesses. In Ireland, a psychoeducation programme called EOLAS (Irish for knowledge) was developed in collaboration with peers, family members, and healthcare workers to help support people with psychosis and their caregivers. [16]. EOLAS is delivered in a manual format and teaches people about psychosis, focusing on the biopsychosocial model, relapse prevention, and how families can support patients [16, 17]. The programme improved the understanding of recovery and communication between healthcare workers and other stakeholders [16]. An e-version of EOLAS has been implemented following the COVID-19 pandemic, and it has shown that it was feasible to use and effective [17]. The findings support the usefulness of digital platforms to deliver health education interventions to support people with mental illness [18, 19].

The effectiveness of ROMHCPs is well documented in the literature [20–23]. However, evidence from the literature indicates that the implementation of these programmes is from mainly high-income countries, mainly from the West [3, 24–26]. Other regions, such as Asia and South America, have adopted ROMHCA despite challenges such as poor implementation [27, 28]. Africa is still behind in delivering mental healthcare services, let alone adopting recovery-oriented mental health care services. Studies from Africa have indicated that the delivery of mental healthcare services is hampered by a lack of mental health policy, overreliance on biomedical approaches, and a general lack of knowledge about recovery-oriented mental health care [29–31].

ROMHCA in Botswana has not yet been implemented and uses the biomedical approach, which does little for people with mental illness at the community level but admits them to mental health institutions [32]. The country operates only one psychiatric hospital, Sbrana Psychiatric Hospital (SPH), with a bed capacity of 300 patients. The provision of mental health care is through the mental health policy of 2003, which serves as a reference for integrating mental health into general health care [33]. SPH is backed by eight mental health units, all with a bed capacity of 10–15 patients. There are 11 psychiatrists in the country, and most are based in private practice. Only two are based at SPH. Psychiatric mental health nurses operate most mental health services with the help of general nurses [32, 34].

With the increasing evidence from international studies on the effectiveness of recovery-oriented mental health services [23, 26, 35], Botswana should also position itself to implement the latest evidence-based care approaches in mental health to support people with mental illness in the community. Following admission, patients are discharged to their families without proper preparation to reintegrate them into society. The mental health service continues to face a shortage of mental healthcare personnel. Nurses are the leading providers of mental health care and mostly see patients first on arrival at mental health facilities [36]. The researchers, who are mental health nurse specialists, have observed that “nurses talk about recovery” in their daily interactions with patients. The meaning attached to recovery is mainly symptom reduction and compliance with treatment. The recovery-oriented practice goes beyond symptom reduction and treatment adherence but calls for patient-centred care [9].

Nurses in Botswana must devise or participate in developing interventions to improve the care of people diagnosed with mental illness. Brooke-Sumner et al. [30] from South Africa have attributed the failure of health workers to implement recovery-oriented mental health services to a lack of understanding and involvement of mental healthcare personnel. In addition, a review of how recovery is understood among ethnic minorities by Sofouli [37] concluded that more work should be carried out on the policy so that cultural minorities can access culturally tailored interventions to address their needs. Therefore, this calls for developing interventions informed by findings from the localised data context to accommodate the uniqueness of different communities. To our knowledge, no study has been conducted in Botswana that solicited nurses' views on how ROMHCP can be developed and implemented. Since most mental healthcare professionals in Botswana are nurses, eliciting their views on developing ROMHCP is essential.

## 2. Methodology

### 2.1. Design

This qualitative study explored nurses' views on developing a recovery-oriented approach. The qualitative method investigates people in their locality and emphasises how the study participants experience and interpret phenomena [38]. Therefore, the design was appropriate since it described the contextual meaning of the participants of how a recovery-oriented mental healthcare programme could be developed and implemented. This study focused on the following questions and further probing statements from the participants to find more meaning in their responses. The study participants were nurses working in mental health facilities, with at least 3 years of experience. It was believed that the participants would be able to use their experience to provide information about the following objective:

1. What are your views on developing a recovery-oriented programme for Botswana?
2. How could a recovery-oriented mental healthcare programme be developed?

### 2.2. Context

The study was conducted in Botswana in four sites offering inpatient mental health care to people with mental illness. Among the four sites was the country's only mental health referral hospital in southeastern Botswana, Lobatse. The other three sites were psychiatric units attached to district hospitals. Of the three sites, one was in the far north west of Botswana Maun, one in central north east Botswana Francistown, and the last in the south west of Botswana, Molepolole. The three mental health units provide care to clients daily, with a bed capacity of 15–20 patients each. However, since they are small units, most of their patients are referred to the main psychiatric hospital, which has a bed capacity of 300.

### 2.3. Population and Sample

The study's population was all the nurses in the four sites actively treating patients with mental illness for at least three years. Thirty-two nurses were purposively selected out of 149 who consented to participate in the study. All the selected nurses consented to participate in the focus group discussions (FGDs). The number of FGDs was determined by data saturation.

### 2.4. Data Collection

Data were collected through online FGDs with nurses with at least three years of experience treating people diagnosed with mental health challenges. Microsoft Teams was used to conduct the FGDs since data were collected during the active phase of the COVID-19 pandemic. Two FGDs had seven participants, and the other two had 8. According to Nyumba et al. [39], six–eight people are ideal for FGDs. The FGDs were preferred for this study due to their ability to produce an interactive atmosphere that allows the participants to produce diverse information quickly from multiple participants [40]. One FGD was conducted per site, and in total, four FGDs were conducted across the four sites.

The steps to recruit nurses and study participants are described below. Through an identified mediator and independent person in each facility, meetings were set, and the purpose of the study was explained to potential participants following the study advertisement. Then, each potential participant was given information about the study and a consent form by an independent person. The nurses who eventually consented to participate voluntarily provided their phone numbers to the researcher through the help of an independent person. All participants consented to be virtually and audio-recorded. K.M.K., a lecturer and a mental health nurse specialist, conducted the FGDs. K.M.K., a PhD student, had undergone training in qualitative data collection methods and had conducted qualitative interviews as a lead investigator in one study before conducting the interviews. K.M.K. was also supervised by two study supervisors experienced in qualitative research methods.

The FGDS were conducted in a private, secure place, and participants participated from their homes. The interviewer used an interview guide pilot tested with the participants from one mental health unit that was not part of the study. The pilot test offered insight into probing to get rich responses. FGDs were conducted in English and Setswana, the local language, allowing the participants to express themselves in the language they felt comfortable in. However, English remains Botswana's official language in workplaces. Besides, all the nurses have undergone intensive training in English. The FGDs lasted between 60 and 80 minutes. The interviewer took notes as the participants expressed their views, summarised each interview, and shared the summary with the participants to validate the content during the interview.

Data saturation was reached at FGD number three although the researcher went on to complete the last FDG to confirm the recurring themes. According to Hennink et al. [41], data saturation refers to a point at which issues become repetitive to the point of redundancy. Furthermore, Saunders et al. [42] conceptualised data saturation as “a matter of identifying redundancy in the data, with no necessary reference linked to these data; saturation appears to be distinct from formal data analysis.” As such, in this study, at the end of the third FGD, it became clear that no new data were identified, confirming that data saturation had been reached. Therefore, the fourth FGD was conducted to confirm the themes.

### 2.5. Data Analysis

Data were analysed using Tesch's eight steps of data analysis [43] as it provides a detailed eight-step guideline for organising unstructured data. First, the interviewer transcribed visual recordings. The interviewer masked the participants' names to ensure confidentiality by using P0–P8. Following data transcription, the researcher (K.M.K.) read all the data carefully to make sense of the transcribed data and notes. Some notes were scribbled on the side of the script. At this point, the researcher generated and wrote topics on the margin of the script. A similar process of note writing was completed for all of the scripts. Then, similar ideas and topics were grouped into columns. Once the ideas were grouped, the researcher took the completed list and revisited the data. Abbreviations were used to code the topics and put them next to the text representing that particular code. That exercise helped to tell if any new codes and categories emerged. The researcher used descriptive words to name and group the topics into categories. At this stage, related topics were grouped to reduce the number of topics. The researchers labelled each category and arranged the codes alphabetically. Data were assembled according to a category it belonged to for preliminary analysis. The repeating data informed the grouping of categories, and irrelevant data were discarded. The researcher then sent raw data to the cocoder for an independent analysis. The cocoder was an experienced mental health specialist qualitative researcher identified and recommended by the study's supervisors.

Subsequently, a meeting was set to discuss the themes and categories, and a consensus was reached. The results were further discussed with the study's participants to ensure that the emerging themes reflected the study context. A virtual meeting was held with the participants and nurses in each site through Microsoft Teams to validate the themes. The participants validated the themes orally and confirmed the descriptions of the sites by raising their hands to confirm that they represented what had been said during the interviews. In addition, the study's supervisors, professors E.M. and S.M., together with experienced qualitative nursing researchers, also checked, validated, and confirmed the themes.

### 2.6. Measures to Ensure Trustworthiness

To ensure the trustworthiness of this qualitative study, the researchers demonstrated thoroughness in credibility, transferability, dependability, authenticity, and confirmability [44]. Credibility was ensured through triangulation and visual recording of the FGDs. Researcher triangulation was achieved by reviewing the visual recordings and comparing them with the emerging themes and subthemes. In addition, credibility was further ensured through prolonged engagement with the study's participants. To achieve that, the interviewer took time, for example, to meet and set an appointment with participants for the FGDs to form and strengthen relationships to build rapport. In addition, the researcher had one virtual meeting with the study's participants to validate the themes and subthemes (member checking). For transferability, the researchers gave a thick description of the context, the study participants, and quotes from the participants. Authenticity was ensured by giving a complete description of the study context and engaging the cocoder to verify the themes. Finally, the researchers documented a detailed description of the research process to ensure dependability and confirmability. Moreover, the research supervisors, EM and SM, monitored the research process throughout data collection and analysis to ensure confirmability. The study was reported based on the consolidated criteria for reporting qualitative research (COREQ) [45].

### 2.7. Ethical Considerations

Fleming and Zegwaard [46] posit that all human research must be approved before data collection to protect the participants and demonstrate good research conduct. Therefore, North-West University Health Research Ethics Committee cleared the study to be conducted. The participants were given information about the study and voluntarily consented to participate. The researcher used codes to protect the anonymity of all those who participated. All participants consented to the visual recording of the data collection procedure. All COVID-19 protocols were adhered to during the phase of study promotion and recruitment of the study participants. The researcher compensated each participant with P200.00 ($12) since they used their data to participate in the online FGDs.

## 3. Results

Thirty nurses participated in the FDGs. The age of the participants ranged from 26 to 53 years. Twenty-five nurses (83.3%) had a diploma in nursing, 14 (46.66%) had a diploma in psychiatric mental health nursing, 3 (10%) had a degree in nursing, and 2 (6.66%) had a master's degree in nursing qualification. Only two 2 (6.66%) nurses were nurse managers. The average experience of working in mental health facilities ranged from 4 to 13 years. All the nurses had more than three years of experience working directly with patients diagnosed with mental illness.

### 3.1. Themes and Subthemes

Participants indicated that Botswana's recovery-oriented mental healthcare programme could be developed and implemented.  Table 1 includes the following themes: (a) “developing and implementing of a recovery-oriented mental healthcare programme,” (b) “elements needed to develop a recovery-oriented mental healthcare programme,” and (c) elements needed to implement recovery-oriented mental healthcare programmes.

### 3.2. Theme 1 Development and Implementation of a Recovery-Oriented Mental Healthcare Programme

#### 3.2.1. Implementation of Recovery-Oriented Mental Health Care Is Possible if It Is Supported

Participants believed that Botswana's mental health facilities could implement the recovery-oriented mental healthcare programme.

F, P4: “I believe this is all possible if we can all come on board, from family, community and leadership levels and prioritise mental illnesses or mental health services as much as we prioritise other health services. So, it can be achieved if we can all come on board.”

In addition, the participants indicated that mental health recovery-oriented care could be developed and implemented if prioritised and well-funded. However, there was a belief that that could only be possible if there was support from the government, hospital managers, and the community.

L, P0: “Yes, it is possible to have a mental health programme in our country, provided mental health is prioritised and supported by the top management.”

S, P1: “I believe it can happen with support.”

### 3.3. Theme 2 Elements Needed to Develop and Implement Recovery-Oriented Mental Healthcare Programmes

Participants in this study believed that a recovery-oriented mental healthcare programme could be developed and implemented and suggested how this could be carried out. They suggested the following subthemes on how a recovery programme could be implemented. Having appropriate policies and up-to-date guidelines and SOPs within and between facilities, including external stakeholders (e.g., pastors and traditional healers) in the recovery plan, the importance of aftercare, and psychoeducation for the community and patients and stigma eradication regarding mental illness in communities are very important.

#### 3.3.1. Proper Policies, Up-to-Date Guidelines, and Standard Operating Procedures (SOPs) within and between Facilities

The participants felt that for a recovery-oriented mental healthcare programme to be successful, there must be appropriate policies, guidelines, and SOPs to guide its development and implementation. They stated as follows:

L, P5: I believe there should be a team in place that can measure whether care rendered to clients is as per the standards according to our SOPs. I believe commitment to quality care in all facilities should be a priority.

M, P6: There must be detailed guidelines on how the client will be assisted starting from admission until discharge.

#### 3.3.2. Inclusion of External Stakeholders (e.g., Pastors and Traditional Healers) in the Recovery Plan

The participants also felt that external stakeholders such as herbalists, traditional doctors, and spiritual leaders should be involved in implementing the mental healthcare programme. Health involves restoring health from all the client's biopsychosocial sphere, including the spiritual needs often left unattended in mental health, as already discussed. They suggested that policies could be developed and implemented to assess the involvement of external stakeholders.

F, P1: “I will give an example if patients want to be seen by a herbalist, spiritualist or traditional doctor in the hospital, there should be assistance to provide holistic care. Someone from the spiritual perspective can pray for them. All stakeholders must come on board to make the patient's recovery possible.”

M, P7: “Spirituality is very important in the recovery of patients. Hospitals should respect the spirituality of patients. Wards and facilities can have policies that regulate at what level patients engage in spirituality.”

#### 3.3.3. The Importance of Aftercare

Furthermore, the results also indicated that the participants felt that the programme should have aftercare and follow-up programmes to cater for the clients once they get discharged from mental health facilities. In addition, there should be a link between the hospital and the community to receive the patients and continuity of care.

S, P4: “It is possible to achieve full recovery of this client by ensuring there are linkages or continuity in the community. If they get discharged from a hospital, there should be continuity of care or after-care to support the patients.”

L, P3: “Sometimes we find that the patients are just discharged into the society without notifying the local clinics of their whereabouts. We must have proper discharge procedures that link the patients to the clinics where they are discharged.”

In addition, one participant emphasised the need for a follow-up plan following the discharge of patients from mental health facilities. For example, the current setup in Botswana needs a better-defined follow-up plan following the discharge of patients from the hospital.

S, P2: “When we discharge patients, there is no follow-up until we see the patient upon readmission. We need personnel that can do follow-ups of patients.”

#### 3.3.4. Psychoeducation for the Community and Patients and Stigma Eradication regarding Mental Illness

Furthermore, there was a great emphasis on implementing a recovery-oriented mental healthcare programme with a strong psychoeducation programme targeting individuals, families, and the community at large on issues of mental health and stigma eradication. They further indicated the need to target the cultural misconceptions about mental illness and its causes by involving cultural leaders in psychoeducation interventions as they greatly influence the community members.

M, P1: “The health care workers should do thorough psychoeducation to the individual, the family and the community. Psychoeducation can include many things regarding the diagnosis that can help the individual and what to expect.”

L, P3: “First, teach the clients and the family about the condition so that the patient can accept themselves and the family accepts the client. That can happen if there is education about the patient's condition and treatment.”

The participants also felt that psychoeducation should be extended to community leaders because of their critical societal roles. There was a belief that because of their influence, they could help dispel the myths and stereotypes that are normally associated with mental illness due to their influence.

S, P4: “Our cultural leaders must be on the same page with modern health. Clients relapse because of the myths surrounding mental health.”

S, P2: “There is a way that culture perceives mental illness. We can reach cultural leaders and the community and teach them how culture and mental illness intersect.”

The issue of stigma towards mental illness also came very strongly. The participants felt that for our setting to be recovery oriented, issues of stigma must be dealt with.

L, P3: “The patient should know about the stigma in society. Sometimes we need to understand that stigma is one of the factors that contribute to patients rejecting treatment. The patient can sometimes commit suicide because of stigma.”

The participants also believed that cultural perspectives and myths surrounding the cause of mental illness influence how patients with a diagnosis of mental illness deal with and facilitate their recovery process.

F, P4: “We still have setbacks regarding mental health issues because of our belief systems. People still hold that mental illness comes as a result of witchcraft. Some believe that you have done something bad to another person and they're trying to fix you. A lot of people have got people with mental illness in their communities and in their families but still fail to accept that mental illness can happen to anyone.”

## 4. Discussion

The study explored how nurses working in four inpatient mental health units in Botswana perceived the development and implementation of a recovery-oriented mental care programme for Botswana. The study used an explorative, descriptive, and qualitative method and FGDs to collect data on nurses' views on the phenomenon.

Participants in this study believed that developing and implementing a recovery-oriented mental healthcare programme could be possible for Botswana nurses to guide the care of people diagnosed with mental illness. The participants further believed that the programme's success could be realised if all stakeholders came on board in support of the programme. Similar findings were reported in a study from Indonesia by Nurhayati et al. [47], where stakeholders indicated that a mental health community recovery programme could be applied in community settings. On the other hand, they averred that the care of people diagnosed with mental illness is still addressed through hospital admissions, where medication is the preferred choice of treatment.

On the important elements in facilitating the development of a recovery-oriented mental healthcare programme, the participants in this study highlighted the importance of having proper policies and guidelines on the proposed programme. In addition, they underscored the need for mental health interventions to be prioritised in terms of funding. The same findings were established in a study by Nurhayati et al. [47]. The participants indicated that there must be supporting policies on the recovery programmes and the availability of funds to ensure their success. A lack of funds for mental health initiatives was reported in a study from Taiwan in [27]. In the study, the staff proposed more resources in implementing recovery-oriented mental health services.

It is crucial for countries implementing a recovery-oriented mental health approach to have proper guidelines and policies to guide its implementation [2, 3, 48, 49]. Policies are effective in enforcing what is on paper. For example, staff and peer workers expressed concern about implementing recovery-oriented care in an organisation that does not support it in terms of having policies and procedures in place [50]. They contended that such a care project was unlikely to succeed. However, nurses in this study believed that the programme could be developed. Nonetheless, the implementation would require policies, procedures, and standards in place.

Other views on essential elements in developing the recovery-oriented mental healthcare programme were the involvement of other stakeholders such as traditional doctors, herbalists, and spiritualists. According to studies conducted in South Africa and Botswana, people diagnosed with mental illness usually seek help from traditional doctors, herbalists, and spiritual healers before they can resort to modern health [31, 51]. In concurrence, nurses in this study suggested that mental health facilities should provide for the spiritual needs of clients in a mental health facility. The participants suggested that patients' spiritual needs could be provided for by having spiritualists, herbalists, and traditional doctors in the facilities to avoid situations where clients might end up overusing the prescriptions they might get from such services, which may be fatal. One participant suggested that the facilities could develop guidelines on how spiritual care could be incorporated and regulated into the mental health services in Botswana.

The importance of aftercare, proper follow-up, and planned home of patients was mentioned by participants as essential in implementing the programme. Participants expressed the need for proper discharge and follow-up channels following discharge from the mental healthcare facility. To corroborate this study's findings, patients in a study conducted in Norway highlighted the importance of follow-up plans for continuity of care [52]. In addition, the participants in the same study suggested that follow-up plans should be communicated on time, be flexible, and include the service users and their families. Findings from Indonesia from a study by Nurhayati et al. [47] also back the study as stakeholders in their study emphasised that home visits were essential to support the recovery of people diagnosed with mental illness. In Botswana, mental health service has a discharge and follow-up plan; however, when patients are discharged, they are not followed and reintegrated into the community. There is a need for this gap to be closed and that could be achieved through developing a functional recovery-oriented mental health programme.

Some recovery-oriented mental healthcare programmes are aftercare programmes that help facilitate patients' recovery and reintegration into the community following discharge from a mental healthcare facility [5, 53]. Being cared for in a community setup has been demonstrated to be effective in helping patients recover from mental illness. Fletcher et al. [53] evaluated the effectiveness of residential recovery-oriented facilities that helped people with severe mental illness from recidivism in Australia. The services effectively provided community-contextualised care that aligned with the recovery principles of empowerment and inclusion in line with the Australian mental health policies of a recovery-oriented approach. In addition, the services were well appreciated by clients as they felt they were supported and valued by staff. The findings were further supported by Heyeres et al. [54], as their study also indicated that residential facilities were recovery focused and complied with the recovery policies set by their government. The findings on the importance of community mental health services in caring for people diagnosed with mental illness could be why nurses in this study felt there should be aftercare and follow-up in place.

Lastly, the participants raised the importance of a functional and practical programme. In their view, the programme should have a psychoeducation programme targeting the staff, the patients, families, and the community. The participants felt a general lack of awareness about mental illness and mental health issues. In addition, stigma towards mental illness and cultural misconceptions about the causes and management of mental illness could hinder the successful implementation of the programme. Similar views were echoed in a study from Tanzania, which reported a general lack of awareness of mental illness and its causes in the community [55].

Furthermore, caregivers in the same study indicated that the community needed to be educated on handling people with mental health problems. The findings are further supported by findings of a study from Taiwan where health workers expressed the need for families and public health education on mental health issues with the hope that once the public is informed, they could be a major contributor to stigma reduction and hence better understanding, leading to the better outcomes for patients [27]. Moreso, the Taiwanese study, emphasised the need for health professionals to be educated on the recovery-oriented mental health approach to enhance their competencies and confidence in caring for clients with mental illness.

A study from Botswana on beliefs on the cause of HIV and mental illness by Becker et al. [32] associated the cause of mental illness with witchcraft. The study further indicated that people diagnosed with mental illness were viewed negatively as dangerous, untrustworthy, and discriminated against in the workplace. The study recommended training and public sensitisation on mental health issues to align rehabilitation services to a recovery-oriented care approach. In addition, a metasynthesis on the beliefs and perceptions about mental health issues by Choudhry et al. [56] associated mental illness with punishment from God. The authors recommended that mental health services develop clinical interventions to address cultural beliefs by encouraging dialogue with traditional leaders and developing strategies for involving them.

In the same vein, the participants in this study suggested that a recovery-oriented mental healthcare programme should include a psychoeducation training component for people with mental illness, families, and mental healthcare personnel on stigma in mental illness. Moreover, a systematic review by Waqas et al. [57] on interventions to reduce stigma in higher education indicated that psychoeducation programmes successfully reduced the self and public stigma of students with mental health problems.

These findings, therefore, emphasise the need to educate the community on mental health issues to dispel stigma and misconceptions about mental illness. Thus, a recovery-oriented mental healthcare programme developed for Botswana should be inclusive and consider elements such as funding, a training plan, the interplay of different stakeholders, and a strong psychoeducation programme to improve its effectiveness.

## 5. Conclusion

This study is a preliminary exploration of factors that could enhance the development and implementation of mental health recovery in Botswana. The study is the first to be conducted in Botswana. It provides insight into how nurses in four inpatient units perceive the implantation of a recovery-oriented approach in mental health services. The study suggested essential factors for consideration for the success of the approach, including the development of mental health policies that support the recovery-oriented approach; revising hospital admission, discharge, and follow-up plans for people diagnosed with mental illness; revitalising the community mental health services; and involvement of other stakeholders such as traditional leaders, herbalists, traditional healers, and spiritualists. In addition, nurses recommended developing health education programmes to reduce the stigmatisation of mentally ill patients in the community. Besides, other influential people such as community cultural leaders such as chiefs and ward men would be targeted for inclusion in the programme. They would help change people's perceptions towards the causes and management of mental illnesses.

### 5.1. Recommendations

Mental health problems remain a burden and a significant public health concern. Unfortunately, mental health programmes remain the least funded among health programmes, especially in low- and middle-income countries [58]. For example, in Botswana, mental health still operates under 1% of the national health budget. The participants in the study recommended that the programme could be developed and implemented if it received funding, involved multiple stakeholders, and had a psychoeducation programme targeting all the major stakeholders, community leaders, mental health personnel, and the patient's families. In a meeting hosted by the WHO in conjunction with the World Bank Group in 2016, Global Mental Health included mental health as one of the sustainable development goals, which underscores the need to prioritise mental health and well-being at all ages [58]. To meet the goal, mental health initiatives should be prioritised in terms of funding.

### 5.2. Relevance to Clinical Practice

The recovery-oriented mental healthcare programme has the potential to benefit people with mental illness in the country. In addition, it would allow nurses to improve their knowledge and skills in managing mental illnesses. Findings might also inspire policy change in Botswana and other countries with limited resources as they move to adopt the recovery-oriented approach and guide its implementation.

## Figures and Tables

**Table 1:** Showing how a recovery-oriented mental healthcare programme can be developed and implemented.

Themes	Subthemes	Categories
(1) Development of a recovery-oriented mental healthcare programme is possible	(1) Implementation of recovery-oriented mental health care is possible if it is supported	(1) Recovery is possible and achievable
		(2) Prioritise mental health initiatives
		(3) Provide funding for mental health

(2) Elements needed to develop and implement recovery-oriented mental healthcare programmes	(1) Having proper policies and up-to-date guidelines and SOPs within and between facilities	(1) Having policies in place
		(2) Have a consistent standard of operations
		(3) Develop guidelines to support the implementation of a recovery programme
	(2) The inclusion of external stakeholders in the recovery plan of patients	(1) Provision of holistic care in mental health services
		(2) Include pastors, traditional healers, herbalists, spiritual healers
		(3) Have policies on the inclusion and regulation of spiritual care
	(3) The importance of aftercare	(1) Have proper discharge and follow-up policies
		(2) Have proper linkages in the community for continuity of care
	(4) Education among the community and the patients themselves (psychoeducation) and stigma eradication	(1) Develop educational programmes for mental health awareness
		(2) Provide education to patients, families, community/society, nurses, and other healthcare workers on mental health issues
		(3) Training plan of nurses on the recovery-oriented mental healthcare approach
		(4) Provide education to society and cultural leaders on mental health issues and cultural misconceptions about mental illness

## Data Availability

The data used to support the findings of this study are available from the corresponding author upon reasonable request.

## Additional Points

*What Does This Paper Contribute to the Clinical Community?*. The paper has the potential to (i) contribute to the transformation of mental health care in Botswana to recovery oriented and (ii) reduce the scarcity of the literature on developing and implementing recovery-oriented mental healthcare programmes in Botswana.
